# COVID-19 Associated Mucormycosis: A Systematic Review from Diagnostic Challenges to Management

**DOI:** 10.3390/diseases9040065

**Published:** 2021-09-22

**Authors:** Farah Yasmin, Hala Najeeb, Aisha Naeem, Kartik Dapke, Rachana Phadke, Muhammad Sohaib Asghar, Syed Muhammad Ismail Shah, Domenico De Berardis, Irfan Ullah

**Affiliations:** 1 Department of Internal Medicine, Dow University of Health Sciences, Karachi 74200, Pakistan; farahasmin972@yahoo.com (F.Y.); ayeshanaeem123@hotmail.com (A.N.); 2 Indira Gandhi Government Medical College, Nagpur 440018, India; kartikdapke4219@gmail.com (K.D.); rachanap05@gmail.com (R.P.); 3 Department of Internal Medicine, Dow University Ojha Hospital, Karachi 74233, Pakistan; sohaib_asghar123@yahoo.com; 4 Department of Internal Medicine, Ziauddin Medical University, Karachi 75600, Pakistan; ismailshah6551@gmail.com; 5 NHS, Department of Mental Health, Psychiatric Service for Diagnosis and Treatment, Hospital “G. Mazzini”, ASL 4, 64100 Teramo, Italy; domenico.deberardis@aslteramo.it; 6 Department of Internal Medicine, Kabir Medical College, Gandhara University, Peshawar 25000, Pakistan; Irfanullahecp2@gmail.com

**Keywords:** severe acute respiratory syndrome coronavirus 2 (SARS-CoV-2), mucormycosis, COVID-19 associated Mucormycosis (CAM), black fungus, hyperglycemia

## Abstract

The coronavirus disease 2019 (COVID-19) outbreak has caused significant destruction, claiming over three million lives worldwide. Post SARS-COV-2 invasion, immunosuppression with hyperglycemia and elevated ferritin levels along with steroidal treatment creates a perfect storm for opportunistic infections. There is increasing evidence of mucormycosis co-infection in COVID-19 patients, during or post-treatment. A worse prognosis, a late diagnosis, and limited guidelines of screening and management of COVID-19 associated mucormycosis have made healthcare professionals fear an epidemic alongside a pandemic. This review geographically reports cases of COVID-19 associated mucormycosis (CAM), evaluates characteristics, clinical manifestations, and outcomes of mucormycosis in COVID-19 active or recovered patients. It further describes preventive strategies and recommendations for optimal management therapy that can be adopted worldwide to curtail an impending threat to the healthcare system.

## 1. Introduction

The novel severe acute respiratory syndrome coronavirus-2 (SARS CoV-2) first reported in Wuhan, China on 21 December 2019 [1,2]. Thereafter, the virus has spread rapidly and affected millions across the globe, and on 11 March 2020, it was finally declared a pandemic. As of June 2021, 172 million people have been affected by this virus with 3.69 million deaths worldwide [3]. The most common presenting complaints of this disease include cough, fever, and dyspnea [4,5]. Extra pulmonary manifestations comprise alteration of taste, olfactory changes, erythematous rashes and urticaria, and even severe neurologic complications like altered consciousness, dizziness, and cerebrovascular events [6,7]. Like SARS-CoV and MERS-CoV, the virus causes lower respiratory tract infection leading to acute respiratory distress syndrome, and eventually ground-glass opacity of the lungs [8]. Due to the severe inflammatory reaction and diffuse alveolar damage, COVID-19 patients experience a decline in their CD-4+ and CD-8+ T cell count, making them susceptible to a wide range of infections, particularly fungal infections [9]. Critically ill patients who were admitted to ICU and required mechanical ventilation or patients with hospitalization of 50 days or longer were more likely to develop fungal co-infections. Therefore, it is immensely crucial to observe such person with COVID-19 because they can develop fungal infections during latter stages of this disease [10].

According to a study conducted in China, Chen et al. found 5% cases of fungal co-infections out of 99 cultures isolated from COVID-19 positive cases, including one case of *Aspergillus flavus*, one case of *Candida glabrata*, and three cases of *C. albicans* [11]. A German study found COVID-19 associated invasive pulmonary aspergillosis (IPA) in five (26.3%) of 19 critically ill patients with moderate to severe ARDS [12]. In Netherlands, there were six patients (19.4%) presumed IPA in 31 ICU patients, of which five were identified as *A. fumigatus* [13]. Amongst all fungal co-infections, the incidence rate of mucormycosis was 0.005 to 1.7 per million population [14].

Mucormycosis (also called zygomycosis) is a serious fungal infection caused by a group of molds called mucoromycetes [15]. The types of fungi that mostly cause mucormycosis include *Rhizopus* spp., *Mucor* spp., *Rhizomucor* spp., *Syncephalastrum* spp., *Cunninghamella bertholletia*, *Apophysomyces* spp., and *Lichtheimia* (formerly *Absidia*) spp. [16]. The *Rhizopus oryzae* is the most common type and responsible for nearly 60% of mucormycosis cases in humans and accounts for 90% of the Rhino-orbital-cerebral (ROCM) form [17].

These fungi live particularly in soil and in decaying organic matter, such as leaves, compost piles, or rotten wood. It is transmitted by coming in contact with the fungal spores in the environment. Mucormycosis may be associated to different clinical manifestations depending on the organs affected. There has been a rise in the number of cases of mucormycosis in patients with COVID-19 worldwide, particularly in India. The reason behind this is the favorable environment in the affected patient that allows the spores to grow. These include hypoxia, high glucose levels due to diabetes or steroid-induced hyperglycemia, acidic medium created by diabetic ketoacidosis or metabolic acidosis, high ferritin levels due to inflammation, and a decreased activity and count of white blood cells along with several underlying conditions that promote the germination of spores and lead to the catastrophic picture of rhino cerebral mucormycosis co-infection with COVID-19 [18]. In this review, we have summarized the pathophysiology, clinical manifestations, diagnosis, and management of mucormycosis associated with COVID-19, and provided a summary of all cases published in the literature.

## 2. Methodology

**Figure 1:**
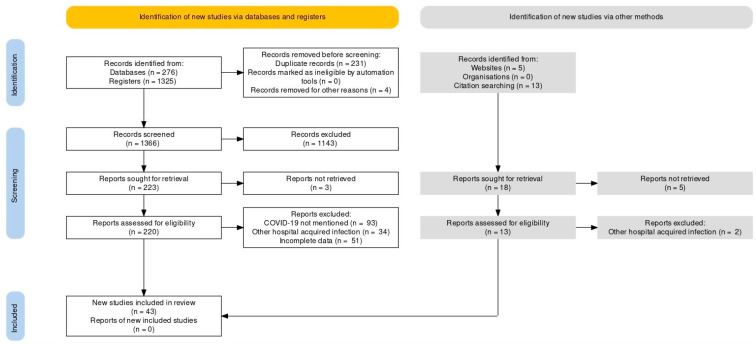
PRISMA Flow Chart.

This systematic review was carried out along the Preferred Reporting Items for Systematic Reviews and Meta-Analyses (PRISMA) guidelines statement [19]. An extensive literature search was conducted using PUBMED/MEDLINE and Google Scholar from inception to June 2021 [20,21,22,23,24,25,26,27,28,29,30,31,32,33,34,35,36,37,38,39,40,41,42,43,44,45,46,47,48,49,50,51,52,53,54,55,56,57,58,59,60,61,62,63,64,65,66,67,68,69,70,71,72,73,74,75,76,77,78,79,80,81,82,83,84,85,86,87,88,89,90,91,92,93]. The following keywords were used as a search string: (“COVID-19” or “SARS-CoV-2”) AND (“Mucormycosis” or “Black Fungus”). A detailed search strategy is given as Supplementary Table S2. Additional search strings included: (“COVID-19” or “SARS-CoV-2”) AND (“Mucormycosis” or “Black Fungus”) AND (“Pathophysiology” OR “Clinical Manifestations” OR “Geographical Distribution” OR “Diagnosis” OR “Management” OR “Treatment”). The search yielded 1619 results, of which 43 studies were included, as shown in Figure 1. Case reports, case series, observational studies and systematic reviews were included in the review. No filters or limitations were applied to the search results. Hand-searching of review articles was performed to extract relevant studies. Relevant studies were imported to Endnote X9 (Clarivate Analytics, Philadelphia, PA, USA) to further remove duplicates.

Inclusion criteria of the study includes published cases of COVID-19 and mucormycosis co-infection. All articles in a language other than the English, studies that did not confirm diagnosis of mucormycosis, websites which reported unpublished cases were excluded from our review.

## 3. Pathophysiology

Previously known as zygomycosis, mucormycosis belongs to the Mucorales group of fungi [16]. The most reported causative agent of Mucorales includes the globally found *Rhizopus, Mucor* spp, followed by *Lichtheimia* spp., *Rhizomucor* spp., *Cunninghamella* spp., *Apophysomyces* spp., and *Saksenaea* spp. [20]. This opportunistic infection, present as spores or hyphae in a hot and humid environment, affects individuals if inhaled or inoculated through cutaneous wounds [21]. Immunocompromised individuals are at the greatest risk of contracting mucormycosis. Predisposing risk factors of mucormycosis include chronic kidney disease, malignancy, neutropenia, increased serum iron, perpetual use of immunosuppressive drugs, and most importantly, diabetes mellitus with ketoacidosis [22].

The immune system has macrophages and neutrophils as the primary defense system against spores. An insufficient immune response allows the spores to germinate into hyphae, and establish infection [22,23]. In diabetic individuals with ketoacidosis, an acidic pH impairs the motility of neutrophils, thus weakening the first barrier to pathogens. Iron, under normal physiologic conditions, remains bound to protein complexes; low pH renders the transferrin system inefficient as it causes unbound iron to circulate in the blood. *Rhizopus* has shown an increased affinity for serum iron at pH < 7.3, allowing it to multiply rapidly in the body [22,23,24]. The increased expression of the cell-surface receptor, glucose-reg-97 (GRP78) under hyperglycemic state causes *R. oryzae* to bind more frequently to endothelial cells in the lungs, brain, and sinuses, and thus, cause damage [25]. Virulence factors of this fungus also include aspartic proteinases which increase disease progression in individuals who have undergone organ-replacement therapy or suffer from comorbidities such as hematological malignancies [26]. Usage of corticosteroids to treat COVID-19 patients suppresses the immune system, and has emerged as a risk factor of mucormycosis, in multiple reported COVID-19 cases [27,28].

Additionally, the incidence of mucormycosis has exasperated as the deadly SARS-CoV-2 alters the immune response. SARS-CoV-2 spreads mainly through droplets and is characterized by the invasion of angiotensin-converting enzyme 2 (ACE2) receptors in humans. ACE2 is present in the lungs, heart, liver, and kidneys. Downregulation of ACE2 by the coronavirus via its spike protein negatively influences the inflammatory-protective renin-angiotensin system [29,30]. The cell-mediated immune response is rendered ineffective as CD 4+ and CD 8+ T-cells, specific for mucormycosis, decrease in COVID-19 patients. The inevitable duo of COVID-associated mucormycosis (CAM) releases a pro-inflammatory cytokine storm of IL-6 and IFN-γ in the infected individual. Lymphopenia thus paves the pathway for opportunistic fungal infections like mucor to resist immune response [29,30,31]. Fungal hyphae produce lesions and thrombi upon entering the blood vessels and invading the walls [32]. The coronavirus infection increases vascular damage to endothelial cells and promotes endotheliitis. Along with vasoconstriction, CAM possesses the capability to necrotize tissues and induce ischemia in organs leading to organ failure [31]. However, this association between the two entities has some confounding factors. For instance, it is possible that the lesions are a consequence of a Delayed-type Hypersensitivity (DTH) reaction, as for other systemic infections, such as Coccidioidomycosis. Further, it is also possible that the fungus disseminates to the skin from the primary infection site, or the cutaneous manifestations are only a consequence of fungal inoculation in the skin by a trauma. Therefore, definitive diagnostic techniques are paramount to early detection and subsequent management.

## 4. Clinical Manifestations

Classification of mucormycosis is based on the site of manifestation. It is enlisted as rhino-cerebral-orbital mucormycosis (RCOM), pulmonary, gastrointestinal, cutaneous, and disseminated mucormycosis, as illustrated in Table 1. RCOM is the prevalent type, and it develops in individuals with diabetic mellitus [14,33,34]. Sporangiospores deposit on the nasal turbinate, and progress through the paranasal sinuses, affecting the maxillary-facial structures, and then disseminates to the brain [35]. RCOM clinically presents as ethmoidal or sphenoidal sinusitis, and leads to cavernous sinus syndrome or internal carotid artery thrombosis. [24,36]. Osteomyelitis of the bony structures of the face results in necrotic ulcers, decreasing functional capabilities of optic and cranial nerves, causing headache and facial pain. Angioinvasion of the palate perforates it and allows the mucor infection to travel through the cribriform plate [22,37]. As it spreads to the orbital region, it degenerates the extra-ocular muscles and manifests as a periorbital syndrome, ptosis, and proptosis in patients. COVID-19 further leads to coagulopathy in the cavernous sinus which under extreme conditions progresses to a permanent loss of vision [35,38,39]. Epistaxis manifests due to the invasion of the brain by RCOM as turbinate bone, and the internal carotid artery become ischemic. Critical co-infection of COVID-19 patients with RCOM, and delayed treatment has led to increasing mortality rates [22,37].

Neutropenia, the use of corticosteroid, and induction chemotherapy increase the risk of pulmonary mucormycosis in individuals [24,34]. Neutropenia and a weakened immune system produce prolonged high-grade fever [24]. SARS-CoV-2 and the *Rhyzomucor* spp. together infect the lungs, the common site of invasion, and induce dyspnea, cough, and airway bleeding [21,37]. Molds of pulmonary mucormycosis affect bronchial airways, and the parenchyma of lungs. Extending as lesions into the chest wall, it poses the threat of cavitation and pericarditis [40]. Additionally, the characteristic and an early diagnostic feature of pulmonary mucormycosis is the reverse-halo sign which appears as consolidation on a Computed Tomography (CT) scan. However, its incidence has been low in COVID-19 patients [41]. Other than cytopenia, certain non-specific laboratory markers which have been associated with COVID-19 related sepsis are also found to be deranged in cases of co-infection with mucormycosis such as lactate dehydrogenase, C-reactive protein, and D-dimer levels, in addition to deranged renal profile [67,82].

Cutaneous mucormycosis results from trauma or burns to the skin, and mostly occurs on the arms and the legs. Surrounding edema and black discoloration at the site of infection leads to gangrene in susceptible hosts. Progressing gradually, nodular lesions appear on the skin [24,42]. While there is one reported case of coinfection in a heart transplant patient [83], COVID-19 itself is associated with vascular lesions, urticaria, and rashes [43]. This amplifies the risk of a possible cutaneous manifestation of mucormycosis. Malnutrition and solid organ transplantation put the individuals at the risk of developing gastrointestinal (GI) mucormycosis in the bowel and the GI tract, specifically the intestines [34,44]. However, the rapid disease progression and the lack of clear clinical signs besides fever delay differential diagnosis from GI diseases [38]. CT scans of isolated cases of GI tract, colonic, and small bowel mucormycosis reveal dilatation of the wall, bleeding, and mass thickening [44]. A rare case of GI mucor in COVID-19 patients confirmed the signs and additionally presented with abdominal tenderness, and bilateral ulceration [45]. Though the rarest type, disseminated mucormycosis has the highest overall mortality in individuals [38]. As a result of this, the only reported case of COVID-19 and disseminated disease was diagnosed after an autopsy [45]. The primary infection can metastasize or undergo a hematogenous spread, producing infarcts in the brain, heart, and spleen. Since it presents with symptoms of underlying comorbidities, diagnosis of disseminated mucormycosis is extremely difficult [34].

## 5. Geographical Distribution

Song et al. identified that critically ill patients in ICUs, and on mechanical ventilation were prone to various fungal infections, as in SARS. Other fungal infections were reported, however, until May 2020, there were no confirmed reports of mucormycosis [46].

**Figure 2:**
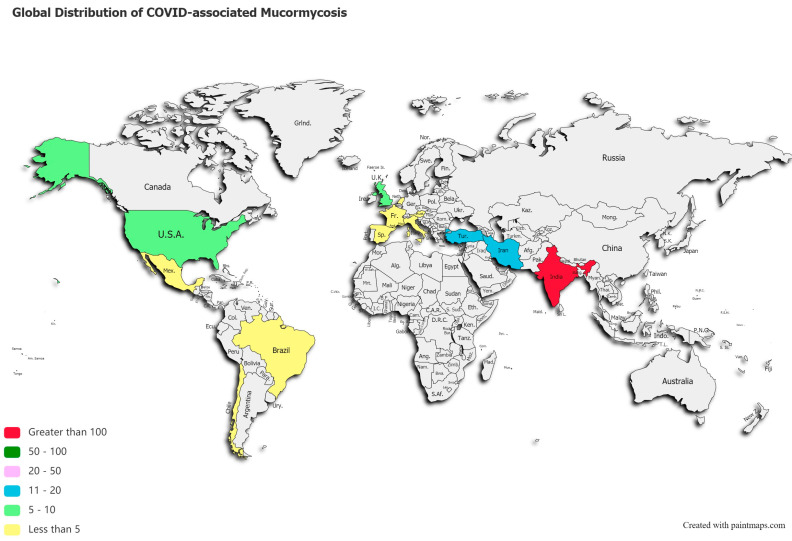
Global distribution of COVID-19 associated mucormycosis.

A post-mortem study conducted between March 2020 and April 2020 from the UK revealed pathological findings in a patient, which upon biopsy, PCR, and DNA extraction confirmed the presence of disseminated mucormycosis [47]. Since then, multiple cases of mucormycosis co-infection in ongoing or post COVID-19 have emerged. Countries amidst the second and the third COVID-19 waves are now overlooking a syndemic sweeping lives globally, as shown in Figure 2. According to a review of published and unpublished studies, CAM has affected 18 countries, including but not limited to India, Pakistan France, Iran, Mexico, Russia, Bangladesh, Brazil, Chile, Czech Republic, Germany, Italy, Kuwait, Lebanon, and Turkey [41]. During the first week of June 2021, India with over 20,000 cases of CAM, remains the hardest-hit country in the world [48].

This systematic review reveals a total of 201 published cases of CAM, and 70 deaths across 13 countries, as shown in Figure 2. India ranked first with a total of 138 cases of the deadly duo, followed by 18 cases in Iran, 12 in Turkey, and 10 reported cases of CAM in the US and UK, each. Supplementary Table S3 stratifies active or post-mortem CAM cases by countries.

## 6. Diagnosis

### 6.1. Culture

Mucorales are saprophytes found in soil and decomposing organic waste. They thrive at temperatures ranging from 25 to 55 °C on most common bacterial (e.g., sheep blood agar, chocolate agar), and fungal culture media (e.g., Sabouraud dextrose agar, inhibitory mold agar, and potato dextrose agar). Mucorales in clinical specimens grow at 37 °C [43], forming fluffy white, grey, or brownish colonies that quickly fill the Petri dish in 1–7 days. Although fungal cultures were shown to be positive in only 50% of cases, recent studies for cutaneous mucormycosis infections have demonstrated a notable rise in culture positivity from 72% to 89% [51]. For direct microscopic detection of Mucorales, a variety of methods (e.g., treatment with 20% potassium hydroxide, Gomori’s methenamine silver staining, hematoxylin, and eosin staining, periodic acid-Schiff staining) can be utilized [51]. Although Mucorales are classically described to exhibit broad (10–50 m), ribbon-like aseptate hyphae with right-angle branching on microscopy, the hyphae are seen to be pauciseptate, and the angle of hyphal branching can vary from 45 to 90° [52]. Furthermore, in the direct microscopic analysis for the Mucorales detection, it is not easy to differentiate Aspergillus hyphae from Mucorales hyphae.

### 6.2. Imaging Techniques

A CT scan is considered better than MRI for fungal infections, especially for visualizing bony destruction. It is also an economically cheaper alternative. It helps visualize any opacification, periosteal thickening, or bony disruption of the sinuses. CT scan has made it possible to detect pulmonary or sinus abnormalities earlier than with traditional sinus and chest radiography. A characteristic reverse halo sign (central ground-glass opacity surrounded by denser consolidation) is observed on CT scans in most patients with mucormycosis. Additional findings like vascular cut-off signs (abrupt termination of a pulmonary artery branch), cavitation, multifocal pneumonia pattern, and peripherally distributed lesions are also seen. The size and extent of lesions point towards the seeding of the infection, most likely to the pleura, and an impending complication like hemorrhage or infarct due to vascular invasion. The development of pulmonary infiltrates in neutropenic patients already indicates tissue damage, angioinvasion, thrombosis, necrosis, bleeding, and edema. Even before localizing symptoms, early CT findings in immunocompromised individuals at high risk for invasive pulmonary mucormycosis can identify pulmonary or Sino nasal lesions in the absence of radiological abnormalities on conventional radiographs. However, the cost and cumulative radiation exposure associated with serial or screening CT scans are some of the commonly disregarded limitations [53].

MRI, on the other hand, is useful for soft tissue imaging and assessing the extent of the disease. A hypotense rim is observed near the lesion edges, which can be attributed to blood products or metals like iron and magnesium accumulated by the fungus although it is common to observe only subtle sinus mucosal thickening or thickening of extraocular muscles and detect no abnormalities in the bones of sinuses even in the presence of the disease clinically in some cases [34].

Orbital invasion, in the case of rhino-cerebral mucormycosis, is followed by rapid involvement of the brain stem and hypothalamus, as seen on MRI. This predisposes the individual to a cavernous sinus infection or internal carotid thrombosis, thus, contributing to higher mortality. Although larger studies are required on this finding, a “black hole” sign has also been observed, which refers to the complete lack of contrast uptake by the lesions [54].

### 6.3. Molecular Diagnosis

The internal transcribed spacer (ITS) region of fungus DNA is the most extensively sequenced, and it is considered as a first-line technique for Mucorales species identification. When used on tissues, molecular-based techniques have acquired acceptability for confirming infection. Methods for detecting Mucorales DNA in blood have yielded encouraging results in terms of detecting the disease sooner and more quickly [93].

## 7. Treatment

Optimal management of mucormycosis can be achieved by coordinated and interdisciplinary efforts by various sectors. Owing to its high mortality, even the slightest clinical suspicion should warrant initiation of antifungal therapy. The management primarily involves a combination of surgical debridement and antifungal therapy. In a multivariate study of 929 reported instances of mucormycosis, Roden et al. found that antifungal medication plus surgery were strongly associated with better survival rates (69%), while death was practically certain (97%) for patients who got no treatment at all [55].

### 7.1. Antifungal Therapy

Amphotericin B is the first-line management of the condition and can contribute significantly to patient outcomes. This was established in a retrospective analysis of 70 patients with mucormycosis who had delayed amphotericin B treatment (initiation of treatment 6 days after diagnosis), which culminated in a nearly twofold increase in death 12 weeks post-diagnosis (83% vs. 49%) [56].

Treatment with amphotericin B is recommended till evident clinical improvement is observed, which usually takes a few weeks. A lipid formulation of IV amphotericin B is mostly used rather than amphotericin B deoxycholate, which is a cheaper and more toxic alternative [57]. Metabolite repletion following amphotericin therapy should also be checked. As noted in a clinical cohort study on 368 patients, routine electrolyte replenishment and intravenous saline hydration contributed to a decline in metabolic abnormalities and renal complications associated with amphotericin B toxicity [58]. Although, amphotericin B has no known efficacy against Cunninghamella and Apophysomyces isolates [93].

Due to its nephrotoxic nature, individuals with impaired renal function should be started on triazoles, namely posaconazole and isavuconazole, which act by inhibiting ergosterol synthesis in the fungal cell membrane [59]. These are broad-spectrum azoles that are effective against mucormycosis agents and are available in both oral and parenteral forms. These are also used as Salvage therapy and as a step-down therapy in patients who can tolerate amphotericin B [60]. Although fungal combination therapy is not indicated in any of the main therapeutic guidelines as of now, larger trials are needed to assess the effectiveness of combination therapy [55].

Isavuconazole is a recently approved drug in the United States and Europe for mucormycosis and has the potential of becoming the mainstay treatment for invasive fungal infections [61]. Like others, it is available in both oral and intravenous forms. A significant improvement in this drug over its predecessor, voriconazole, is the lack of cyclodextrins which are responsible for nephrotoxicity. An extended half-life also makes a once-daily regimen possible [62]. Multicentric clinical studies have also found that there is no difference in mortality rates between patients treated with Amphotericin B or isavuconazole [51]. A significantly improved survival rate has also been reported in DKA mice infected with *Rhizopus* spp. and combination therapy with caspofungin plus Amphotericin B Lipid Complex (ABLC) as compared to monotherapy or placebo. Liposomal Amphotericin B plus either micafungin or anidulafungin has resulted in better outcomes in disseminated mucormycosis as well. If LFAB-echinocandin treatment for mucormycosis is considered in the current scenario, it should be administered at doses approved by the US Food and Drug Administration (FDA) [62].

### 7.2. Surgical Debridement

Angioinvasion and blood vessel thrombosis can result in poor drug bioavailability to the infection site. Patients should be prepared and prioritized for surgery, even with the slightest suspicion of mucormycosis. Surgically debridement of the affected site has been shown to contribute significantly to decreasing mortality [61]. An MRI/CT guided endoscopic sinus approach should be employed to extract the affected tissue. Rapid invasion of the orbits (<72 h) should be managed by orbital exenteration and aggressive debridement of the paranasal sinuses. Patients should be continued on IV amphotericin B followed by step-down therapy. Refractory cases should be managed with triazoles. In severely immunocompromised individuals, efforts should be made to rectify the immunosuppression first and treat it with antifungal medication later [57]. Improvements should be followed up with repeat imaging and conservative management.

### 7.3. Adjunctive Therapies

In patients with hematologic abnormalities, any effort to reverse neutropenia should be made, whether using hematopoietic growth agents or, in certain circumstances, white cell infusions. If feasible, individuals with corticosteroid-induced immunosuppression, such as those with autoimmune disorders, should be weaned or switched to non-steroidal treatment. Antiretroviral medication should be initiated for HIV/AIDS patients to help with their immunity. For individuals with uncontrolled diabetes and/or ketoacidosis, rigorous glycemic management is critical. Although they warrant more studies, iron chelator therapies also remain to be a plausible therapy for patients with DKA. By chelating with unbound iron in patients with diabetes, especially ketoacidosis, they could potentially benefit in conditions with iron overload. Physicians must also prioritize the management of any other existing comorbidities [63].

Hyperbaric oxygen (HBO) therapy has also been seen to be an adjunctive therapy along with the others. The higher oxygen pressure that is provided is known to improve neutrophil functioning and increase AMB activity by correcting acidosis. Finally, increased oxygen pressure reduces fungus development by inhibiting spore germination and speeds wound healing. As a result, HBO treatment for mucormycosis has been recommended as an addition to surgical and antifungal therapy [55]. In such circumstances, health professionals can intervene in a variety of ways to improve the clinical outcome. Due to the difficulties in detecting these infections, a considerable burden of death can be linked to the late beginning of antifungal therapy. Currently, blood culture is the most common method of diagnosis. Because of their low sensitivity, cultures take a long time to produce a result. Enzyme-Linked Immunosorbent Assays (ELISA), which are faster and more precise, should be used in conjunction with cultures to identify the species efficiently as well as monitor the resistance acquisition. Any clinical suspicion of fungal infection should be investigated, and antifungal medication started right away.

## 8. Recommendations

To ensure the best outcomes, healthcare providers must also keep an eye out for linked co-morbidities and create individual-specific treatment regimens and drug dosages. There needs to be more awareness among the public regarding mucormycosis and common individual precautions against it. This is a highly important issue with long-term consequences, and the government must prioritize and devise measures to tackle the endemic by providing enough resources before it becomes an epidemic.

## 9. Limitations

The current study had few limitations. Since it is a systematic review, there are no studies available that establish a causal effect relationship between COVID-19 and mucormycosis. It appears to be a number of factors that may contribute to superimposed mucormycosis in individuals co-infected with COVID-19. However, since majority of literature is based on findings of case reports/series which are subjected to publication bias and considerable heterogeneity. So, they may be an underrepresentation of the burden of disease since establishing a microbiological or histopathological diagnosis is difficult. While the lack of a reliable laboratory marker may also hinder the true estimation of mucormycosis co-infection incidence in COVID-19, it also compounded the effect of treatment in these patients.

## 10. Conclusions

Mucormycosis is an emerging problem in individuals with COVID-19 as well as the healed cases and denotes a poor prognosis. The multisystem involvement and rapid progression associated with the disease warrants additional medical intervention and must be given priority. Supplementary Table S1 provides a summary of case distribution, diagnostic procedures, treatments, outcomes, and death in published literature until June 2021.

## Figures and Tables

**Table 1:** Summary of mucormycosis clinical manifestations in COVID-19 patients.

Type	Pathogenesis	Clinical Manifestation	Risk Factors
Rhino-cerebral mucormycosis	Spores invade sinuses, cribriform plates, and through the cavernous sinus.	Infects the sinuses and spreads to the brain. Destroys maxillary-facial structures and causes ptosis, proptosis, and permanent vision loss [37].	Common in patients with uncontrolled diabetes [49] and kidney transplant.
Pulmonary mucormycosis	Spread of fungal infection through the bloodstream.	Destroys bronchial airways, causes dyspnoea, tracheal invasions of the lungs, and a reverse halo sign on CT scan.	Patients with cancer, post-transplant immunosuppressive therapy [21].
Gastrointestinal mucormycosis	Inhaling spores that invade the GI tract.	Fever, bowel, and per rectal bleed [45,50].	Consistent use of broad-spectrum antibiotics, malnutrition, and neutropenia.
Cutaneous mucormycosis	Direct inoculation of skin through site of trauma or thermal burns.	Black discolouration and lesions on the skin.	Skin trauma such as surgery or burns. It does not involve an impaired immunological response.
Disseminated mucormycosis	Occurs when the infection spreads through the bloodstream to another part of the body	Commonly affects the brain, but also other organs such as the spleen, heart, and skin.	Iron overload, neutropenia, suppressed immune system [24].

## Data Availability

No new data were created or analyzed in this study. Data sharing is not applicable to this article.

## Supplementary Materials

The following are available online at https://www.mdpi.com/article/10.3390/diseases9040065/s1, Table S1: Characteristics of studied included in the systematic review, Table S2: Detailed search strategy, Table S3: Total number of cases of COVID-associated mucormycosis (CAM) by countries.
