# Supplementary material for: COVID-19 Associated Mucormycosis: A Systematic Review from Diagnostic Challenges to Management
Source: Diseases. 2021 Sep 22;9(4):65. doi: 10.3390/diseases9040065 (PMC8544552; doi:10.3390/diseases9040065)
Supplement: Supplementary file 1 [file diseases-09-00065-s001.zip › diseases-1334109-supplementary.pdf]

Online Supplementary Material

Supplementary Table S1: Characteristics of studied included in the systematic review.

First Author	Country	Time Period of Study	Type of Study	Number of People Affected	Number of Deaths	Diagnosis	Treatment	Outcome
**Satish et al** [1]	India	March 2020 - Dec 2020	Retrospective Chart Review	25	2	KOH staining of Nasal cavity crusts, CT scan	IV amphotericin (50 mg/day). Debridement once stable	Surgical debridement in 20 people, 5 patients went against medical advice.<br>1 patient presented one month later with vision loss.<br>2 patients died on follow up.
**Ravani et al** [2]	India	Sept 2020- mid March 2021	Retrospective cohort study	31	3	CT imaging	IV Amphotericin B	3 patients died, 28 patients recovered and were alive on follow up
**Sharma et al** [3]	India	April 2021	Observational study	23	0	CT imaging	Surgical debridement	All recovered except 2 who were lost to follow up
**Singh et al** [4]	UK, USA, Mexico, Iran, Brazil, France, Italy, Turkey, Austria	May 2021	Systematic Review	101	31	N/A	N/A	N/A
**Mehta et al** [5]	India	Sept 2020	Case Report	1	1	MRI, nasal biopsy	IV meropenem (1 gm thrice daily), Vancomycin (1gm twice daily), Amphotericin B (0.5mg/kg/day), cessation of steroid therapy	Died on 6th day of admission
**Werthman-Ehrenreich et al** [6]	USA	April 2021	Case Report	1	1	Face CT scan, nasal swab culture, MRI of brain	Vancomycin, Piperacillin-tazobactem, Amphotericin B	Died on day 26 of hospitalization.
**Sarkar et al** [7]	India	April 2021	Letter to Editor	10	4	KOH staining, nasal biopsy,	Amphotericin B,<br>4 patients received Remdesivir	4 patients died within 1 month of diagnosis, 5 had

								irreversible vision loss, 1 had favourable outcome
**Revannavar et al** [8]	India	April 2021	Case Report	1	0	Functional endoscopic sinus surgery (FESS), Nasal biopsy, culture	Amphotericin B, aspirin	Showed favourable outcome
**Karimi-Galougahi et al** [9]	Iran	March 2021	Case Report	1	0	Sino-nasal endoscopy	Insulin, systemic anti-fungal, surgical debridement	Exenteration of the eye, the patient survived
**Mekonnen et al** [10]	USA	Nov 2020	Case Report	1	1	CT angiography, nasal endoscopic biopsy, Fungal culture	IV vancomycin, cefepime, Liposomal Amphotericin B, glucose management	Died on day 31 of hospitalization
**Do Monte Junior et al** [11]	Brazil	Nov 2020	Case Report	1	1	Chest CT, esophago-gastro-duodenoscopy	Ceftriaxone, azithromycin, oseltamivir, hydrocortisone	Died 1 week after hospitalization
**Garg et al** [12]	India	Feb 2021	Case Report & Systematic Review	8 (1 index case)	7	Sputum fungal culture	Liposomal Amphotericin B (3mg/kg)	Index case discharged 54 days after hospitalization and received Amphotericin B on an outpatient basis
**Alekseyev et al** [13]	USA	March 2021	Case Report	1	0	CT scan and MRI	Multiple surgical debridements, IV abelcet, IV heparin	Discharged to complete course of coumadin and Abelcet
**Johnson et al** [14]	USA	June 2021	Case Report	1	1	Bronchioalveolar lavage (BAL), fungal culture	IV Liposomal amphotericin B, IV voriconazole, IV vancomycin, IV ceftriaxone	Discharged to long term acute care facility
**Veisi et al** [15]	Iran	April 2021	Case Report	2	1	Nasal endoscopic biopsy	Amphotericin B, Endoscopic debridement	1 patient died, 1 was managed successfully
**Maini et al** [16]	India	May 2021	Case Report	1	0	FESS, MRI	IV Fluconazole, IV Amphotericin B	Recovered with minimum deformity
**Saldanha et al** [17]	India	April 2021	Case Report	1	0	Nasal endoscopy, CT scan	Endoscopic surgery, Amphotericin B (25 mg/day)	Discharged with left eye vision loss
**Pasero et al** [18]	Italy	Dec 2020	Case Report	1	1	Bronchial aspirate culture, CT scan, sinus biopsy	Amphotericin B (5mg/kg), surgical debridement, Isavuconazole	Died on day 62 of ICU admission
**Zurl et al** [19]	Austria	Feb 2021	Case Report	1	1	CT scan, post-mortem lung tissue biopsy	Tocilizumab, high dose glucocorticoids	Died on day 24 of symptom onset
**Hoenigl et al** [20]	India, USA, Pakistan	April 2021	Systematic Review	80	39	Radiologic findings, positive culture, or PCR	Amphotericin B	39 patients died, 19 lost vision,

	France, Iran, Mexico, Russia, Bangladesh, Brazil, Chile, Czech Republic, Germany, Italy, Kuwait, Lebanon Turkey						Posaconazole, Isavuconazole in combination, as salvage or monotherapy	Surgery improved outcome
**Krishna et al** [21]	UK	April 2020	Case Report	1	1	CT pulmonary angiography, Tracheal aspirate culture, urine culture, Fungal biomarkers, PCR, autopsy	Steroids, continuous venovenous hemodiafiltration, therapy to meropenem and teicoplanin	Died after 20 days of ICU stay
**Arana et al** [22]	Spain	April & Nov 2020	Case Report	2	0	Facial CT, endoscopic evaluation,	Amphotericin B and an azole, tacrolimus, and posaconazol, surgical debridement, fasciotomy	Recovery in 5 months for patient 1, and in 3 months for patient 2
**Nehara et al** [23]	India	Nov – Dec 2020	Case Series	5	2	CT paranasal sinus, MRI Brain and orbit, Lactophenol cotton blue and KOH mount	liposomal amphotericin B (5mg/kg/day)	2 patients died, 2 patients had a full recovery, 1 patient lost an eye
**Krishna et al** [24]	India	April 2021	Case Reports	2	0	CBCT, CT scan, surgical resection	IV liposomal Amphotericin B 5 mg/kg/day, Itraconazole 200 mg, IV liposomal Amphotericin B 250 mg, Posaconazole 300 mg	Full recovery
**Rao et al** [25]	India	June 2021	Case Report	1	0	MRI, post-contrast scans, MR angiography, endoscopic-guided deep nasal swab	intravenous liposomal amphotericin	Partial recovery with loss of direct and consensual light reflex
**Khatri et al** [26]	USA	Feb 2020	Case report and Review	1	1	CT chest	IV liposomal amphotericin B 550 mg/d and posaconazole 300 mg/d, debridement	The patient did on the 175th day of hospital stay
**Waizel-Haiat et al** [27]	Mexico	Feb 2021	Case Report	1	1	contrast-enhanced CT, Rhinoscopy	Surgical management	Death due to organ failure
**Kanwar et al** [28]	USA	Nov 2020	Case Report	1	1	CT chest, blood, and sputum cultures	intravenous (IV) vancomycin, piperacillin-tazobactam	The patient succumbed to cardiac arrest

**Sen et al** [29]	India	Aug 2020 – Dec 2020	Retrospective Study	6	0	sinus biopsy, deep nasal swab, MRI, CT scan	IV liposomal amphotericin B, posaconazole and surgical debridement	Full recovery of all patients at follow-up
**Meshram et al** [30]	India	May 2021	Letter to the editor	2	2	MRI-PNS, bronchoalveolar lavage with biopsy	Maxillectomy, IV amphotericin B	Both patients succumbed to death
**Miishra et al** [31]	India	Aug 2020 – Dec 2020	Retrospective Case Review	10	5	CT PNS and MRI	Local debridement, IV amphotericin B, FESS	1 patient was lost to follow-up, 4 patients died, 4 recovered
**Moorthy et al** [32]	India	May 2020 – Dec 2020	Retrospective multi-centric study	17	7	CT, MRI of the face, orbits, and brain, KOH test, sinus endoscopy	surgical debridement, maxillectomy, liposomal amphotericin therapy 3–5 mg/kg	10 patients recovered, 7 patients died of which 1 was lost to follow-up
**Hanley et al** [33]	UK	March – April 2020	Post-mortem study	9	9	Full autopsies, percutaneous biopsy sampling	N/A	Death
**Dallalzadeh et al** [34]	USA	Jan 2021	Case Report	2	2	Fundoscopic exam, MRI, culture, CT scan	Intravenous amphotericin, isovuconazole, and micafungin, lateral canthotomy, and cantholysis	Died on day 4 of treatment
**Placik et al** [35]	USA	Aug 2020	Case Report	1	1	Intraoperative samples, CT chest	amphotericin B, surgical resection	Died on day 21
**Bellanger et al** [36]	France	Dec 2020	Case Report	1	1	Respiratory samples, CT	liposomal amphotericin B	Died after 40 days of admission
**Sargin et al** [37]	Turkey	Jan 2021	Case Report	1	1	biopsy and culture	Liposomal amphotericin B, debridement, tracheostomy, Colimicin treatment	Died on the 10th day of hospitalization
**Pakdel et al** [38]	Iran	June 2021	Cross-sectional	15	7	Biopsy, MRI, CT scan	IV amphotericin B liposomal, oral Posaconazole, IV Caspofungin, sinus debridement	7 patients died, 8 recovered
**Buil et al** [39]	Netherlands	Dec 2020 – May 2021	Case series	4	3	CT scan, sputum cultures, lung biopsy	liposomal amphotericin B, Voriconazole, Posaconazole, Surgical debridement	3 patients died in ICU, 1 is undergoing antifungal treatment in ICU
**Bayram et al** [40]	Turkey	March 2020 - Dec 2020	Prospective observational	11	7	CT scan/MRI	intravenous and retrobulbar liposomal amphotericin B, radical debridement	4 patients recovered, 7 died
**Rabagilati et al** [41]	Chile	May– July, 2020	Retrospective cohort	2	1	CT scan, chest x-ray	Liposomal amphotericin B	1 patient died; 1 patient recovered

**Bhavani et al** [42]	India	March 2021	Case Report	1	N/A	CT scan/MRI, biopsy	N/A	N/A
**Khan N et al** [43]	USA	Nov 2021	Case Report	1	1	CT scan	voriconazole 250 mg IV, piperacillin/tazobactam 4.5 g IV	Died on day 17 of hospital admission

Supplementary Table S2: Detailed search strategy

**PubMed (276)**	("covid 19"[All Fields] OR "covid 19"[MeSH Terms] OR "covid 19 vaccines"[All Fields] OR "covid 19 vaccines"[MeSH Terms] OR "covid 19 serotherapy"[All Fields] OR "covid 19 serotherapy"[Supplementary Concept] OR "covid 19 nucleic acid testing"[All Fields] OR "covid 19 nucleic acid testing"[MeSH Terms] OR "covid 19 serological testing"[All Fields] OR "covid 19 serological testing"[MeSH Terms] OR "covid 19 testing"[All Fields] OR "covid 19 testing"[MeSH Terms] OR "sars cov 2"[All Fields] OR "sars cov 2"[MeSH Terms] OR "severe acute respiratory syndrome coronavirus 2"[All Fields] OR "ncov"[All Fields] OR "2019 ncov"[All Fields] OR (("coronavirus"[MeSH Terms] OR "coronavirus"[All Fields] OR "cov"[All Fields]) AND 2019/11/01:3000/12/31[Date - Publication]) OR ("sars cov 2"[MeSH Terms] OR "sars cov 2"[All Fields] OR "sars cov 2"[All Fields])) AND ("mucormycosis"[MeSH Terms] OR "mucormycosis"[All Fields] OR "mucormycoses"[All Fields] OR (("african continental ancestry group"[MeSH Terms] OR ("african"[All Fields] AND "continental"[All Fields] AND "ancestry"[All Fields] AND "group"[All Fields]) OR "african continental ancestry group"[All Fields] OR "black"[All Fields] OR "african americans"[MeSH Terms] OR ("african"[All Fields] AND "americans"[All Fields]) OR "african americans"[All Fields] OR "blacks"[All Fields] OR "blackness"[All Fields]) AND ("fungi"[MeSH Terms] OR "fungi"[All Fields] OR "fungus"[All Fields] OR "fungis"[All Fields] OR "microbiology"[MeSH Subheading] OR "microbiology"[All Fields])))
**PMC NCBI (1325)**	(("COVID-19"[All Fields] OR "COVID-19"[MeSH Terms] OR "COVID-19 Vaccines"[All Fields] OR "COVID-19 Vaccines"[MeSH Terms] OR "COVID-19 serotherapy"[All Fields] OR "COVID-19 Nucleic Acid Testing"[All Fields] OR "covid-19 nucleic acid testing"[MeSH Terms] OR "COVID-19 Serological Testing"[All Fields] OR "covid-19 serological testing"[MeSH Terms] OR "COVID-19 Testing"[All Fields] OR "covid-19 testing"[MeSH Terms] OR "SARS-CoV-2"[All Fields] OR "sars-cov-2"[MeSH Terms] OR "Severe Acute Respiratory Syndrome Coronavirus 2"[All Fields] OR "NCOV"[All Fields] OR "2019 NCOV"[All Fields] OR (("coronavirus"[MeSH Terms] OR "coronavirus"[All Fields] OR "COV"[All Fields]) AND 2019/11/01[PubDate] : 3000/12/31[PubDate])) OR ("sars-cov-2"[MeSH Terms] OR "sars-cov-2"[All Fields] OR "sars cov 2"[All Fields])) AND (("mucormycosis"[MeSH Terms] OR "mucormycosis"[All Fields]) OR (("african continental ancestry group"[MeSH Terms] OR ("african"[All Fields] AND "continental"[All Fields] AND "ancestry"[All Fields] AND "group"[All Fields]) OR "african continental ancestry group"[All Fields] OR "black"[All Fields] OR "african americans"[MeSH Terms] OR ("african"[All Fields] AND "americans"[All Fields]) OR "african americans"[All Fields]) AND

	("fungi"[MeSH Terms] OR "fungi"[All Fields] OR "fungus"[All Fields]))) AND ("0001/01/01"[PubDate] : "2021/06/30"[PubDate])

Supplementary Table S3: Total number of cases of COVID-associated mucormycosis (CAM) by countries.

Country	Number of CAM cases
India	138
Iran	18
Turkey	12
USA	10
UK	10
Netherlands	4
Chile	2
Spain	2
Austria	1
France	1
Brazil	1
Mexico	1
Italy	1

References

1. Satish, D.; Joy, D.; Ross, A.; Balasubramanya. Mucormycosis coinfection associated with global COVID-19: a case series from India. *Int. J. Otorhinolaryngol. Head. Neck. Surg* **2021**, *7*, 815; doi: 10.18203/issn.2454-5929.ijohns20211574.
2. Ravani, S.A.; Agrawal, G.A.; Leuva, P.A.; Modi, P.H.; Amin, K.D. Rise of the phoenix: Mucormycosis in COVID-19 times. *Indian. J. Ophthalmol* **2021**, *69*, 1563–8; doi: 10.4103/ijo.IJO_310_21.
3. Sharma, S.; Grover, M.; Bhargava, S.; Samdani, S.; Kataria, T. Post coronavirus disease mucormycosis: a deadly addition to the pandemic spectrum. *J. Laryngol. Otol* **2021**, *135*, 442–7; doi: 10.1017/S0022215121000992.
4. Singh, A.K.; Singh, R.; Joshi, S.R.; Misra, A. Mucormycosis in COVID-19: A systematic review of cases reported worldwide and in India. *Diabetes. Metab. Syndr. Clin. Res. Rev* **2021,** *15*, 102146; doi: 10.1016/j.dsx.2021.05.019.
5. Mehta, S.; Pandey, A. Rhino-Orbital Mucormycosis Associated With COVID-19. *Cureus* **2020,** 12, e10726; doi: 10.7759/cureus.10726.
6. Werthman-Ehrenreich, A. Mucormycosis with orbital compartment syndrome in a patient with COVID-19. *Am. J. Emerg. Med* **2021**, *42*, e5-e8; doi: 10.1016/j.ajem.2020.09.032.

7. Sarkar, S.; Gokhale, T.; Choudhury, S.; Deb, A. COVID-19 and orbital mucormycosis. *Indian. J. Ophthalmol* **2021**, *69*, 1002–1004. doi: 10.4103/ijo.IJO_3763_20.
8. Revannavar, S.M.; Supriya, P.S.; Samaga, L.; Vineeth V.K. COVID-19 triggering mucormycosis in a susceptible patient: a new phenomenon in the developing world? *BMJ. Case. Rep* **2021,** *14*, 241663. doi: 10.1136/bcr-2021-241663.
9. Karimi-Galougahi, M.; Arastou, S.; Haseli, S. Fulminant mucormycosis complicating coronavirus disease 2019 (COVID-19). *Int. Forum. Allergy. Rhinol* **2021**, *11*, 1029–1030; doi: 10.1002/alr.22785.
10. Mekonnen, Z.K.; Ashraf, D.C.; Jankowski, T.; Grob, S.R.; Vagefi, M.R.; Kersten, R.C.; et al. Acute Invasive Rhino-Orbital Mucormycosis in a Patient with COVID-19-Associated Acute Respiratory Distress Syndrome. *Ophthal. Plast. Reconstr. Surg* **2021**, *37*, E40–2; doi: 10.1097/IOP.0000000000001889.
11. Monte Junior, E.S.D.; Santos, M.E.L.D.; Ribeiro, I.B.; Luz, G.O.; Baba, E.R.; Hirsch, B.S.; et al. Rare and fatal gastrointestinal mucormycosis (Zygomycosis) in a COVID-19 patient: A case report. *Clin Endosc* **2020**, *53*, 746–749; doi: 10.5946/ce.2020.180.
12. Garg, D.; Muthu, V.; Sehgal, I.S.; Ramachandran, R.; Kaur, H.; Bhalla, A.; et al. Coronavirus Disease (Covid-19) Associated Mucormycosis (CAM): Case Report and Systematic Review of Literature. *Mycopathologia* **2021**, *186*, 289–98; doi: 10.1007/s11046-021-00528-2
13. Alekseyev, K.; Didenko, L.; Chaudhry, B. Rhinocerebral Mucormycosis and COVID-19 Pneumonia. *J. Med. Cases* **2021,** *12*, 85; doi: 10.14740/jmc3637.
14. Johnson, A.K.; Ghazarian, Z.; Cendrowski. K.D/; Persichino, J.G. Pulmonary aspergillosis and mucormycosis in a patient with COVID-19. *Med. Mycol. Case. Rep* **2021**, *32*, 64–7; doi: 10.1016/j.mmcr.2021.03.006.
15. Veisi, A.; Bagheri, A.; Eshaghi, M.; Rikhtehgar, M.H.; Kanavi, M.R.; Farjad, R. Rhino-orbital mucormycosis during steroid therapy in COVID-19 patients: A case report. *Eur. J. Ophthalmol* **2021,** 11206721211009450**;** doi: 10.1177/11206721211009450.
16. Maini, A.; Tomar, G.; Khanna, D.; Kini, Y.; Mehta, H.; Bhagyasree, V. Sino-orbital mucormycosis in a COVID-19 patient: A case report. Int. J. Surg. Case. Rep **2021**, *82*, 105957; doi: 10.1016/j.ijscr.2021.105957.
17. Saldanha, M.; Reddy. R.; Vincent, M.J. Title of the Article: Paranasal Mucormycosis in COVID-19 Patient. *Indian. J. Otolaryngol. Head. Neck. Surg* **2021**, 1–4; doi: 10.1007/s12070-021-02574-0.
18. Pasero, D.; Sanna, S.; Liperi, C.; Piredda, D.; Branca, GP.; Casadio, L.; et al. A challenging complication following SARS-CoV-2 infection: a case of pulmonary mucormycosis. *Infection* **2020**; doi: 10.1007/s15010-020-01561-x.
19. Zurl, C.; Hoenigl, M.; Schulz, E.; Hatzl, S.; Gorkiewicz, G.; Krause, R.; et al. Autopsy proven pulmonary mucormycosis due to Rhizopus microsporus in a critically Ill COVID-19 patient with underlying hematological malignancy. *J. Fungi* **2021**, *7*, 88; doi: 10.3390/jof7020088.
20. Hoenigl M, Seidel D, Carvalho A, Rudramurthy SM, Arastehfar A, Gangneux JP, et al. The Emergence of COVID-19 Associated Mucormycosis: Analysis of Cases From 18 Countries. *SSRN Electron J* **2021**, [Preprint]; Available from: https://www.ssrn.com/abstract=3844587

21. Krishna, V.; Morjaria, J.; Jalandari, R.; Omar, F.; Kaul, S. Autoptic identification of disseminated mucormycosis in a young male presenting with cerebrovascular event, multi-organ dysfunction and COVID-19 infection. *IDCases* **2021**, *25*, e01172; doi: 10.1016/j.idcr.2021.e01172.
22. Arana, C.; Cuevas Ramírez, R.E.; Xipell, M.; Casals, J.; Moreno, A.; Herrera, S.; et al. Mucormycosis associated with covid19 in two kidney transplant patients. *Transpl. Infect. Dis* **2021**, e13652; DOI: 10.1111/tid.13652.
23. Nehara, H.R.; Puri, I.; Singhal, V.; IH S, Bishnoi BR, Sirohi P. Rhinocerebral mucormycosis in COVID-19 patient with diabetes a deadly trio: Case series from the north-western part of India. *Indian. J. Med. Microbiol* **2021**, *39*, 380–3; doi: 10.1016/j.ijmmb.2021.05.009.
24. Sai Krishna, D.; Raj, H.; Kurup, P.; Juneja, M. Maxillofacial Infections in Covid-19 Era—Actuality or the Unforeseen: 2 Case Reports. *Indian. J. Otolaryngol. Head. Neck. Surg* **2021**, 1–4; doi: 10.1007/s12070-021-02618-5.
25. Rao, R.; Shetty, A.P.; Nagesh, C.P. Orbital infarction syndrome secondary to rhino-orbital mucormycosis in a case of COVID-19: Clinico-radiological features. *Indian. J. Ophthalmol* **2021,** *69*, 1627–30; doi: 10.1007/s12070-021-02618-5.
26. Khatri, A.; Chang, K-M.; Berlinrut, I.; Wallach, F. Mucormycosis after Coronavirus disease 2019 infection in a heart transplant recipient – Case report and review of literature. *J. Med. Mycol* **2021**, *31*, 101125; doi: 10.1016/j.mycmed.2021.101125.
27. Waizel-Haiat, S.; Guerrero-Paz, J.A.; Sanchez-Hurtado, L.; Calleja-Alarcon, S.; Romero-Gutierrez, L. A Case of Fatal Rhino-Orbital Mucormycosis Associated With New Onset Diabetic Ketoacidosis and COVID-19. *Cureus* **2021** *13*, e13163; doi: 10.7759/cureus.13163.
28. Kanwar, A.; Jordan, A.; Olewiler, S.; Wehberg, K.; Cortes, M.; Jackson, B.R. A fatal case of rhizopus azygosporus pneumonia following covid-19. *J. Fungi* **2021**, *7*:1–6; doi: 10.3390/jof7030174.
29. Sen, M.; Lahane, S.; Lahane, T.P.; Parekh, R.; Honavar, S.G. Mucor in a Viral Land: A Tale of Two Pathogens. *Indian. J. Ophthalmol* **2021,** *69*:244–52; doi: 10.4103/ijo.IJO_3774_20.
30. Meshram, H.S.; Kute, V.B.; Chauhan, S.; Desai, S. Mucormycosis in post-COVID-19 renal transplant patients: A lethal complication in follow-up. *Transpl. Infect. Dis* **2021**, e13663; doi: 10.1111/tid.13663.
31. Mishra, N.; Mutya, V.S.S.; Thomas, A.; Rai, G.; Reddy, B.; Mohanan A.A.; et al. A case series of invasive mucormycosis in patients with COVID-19 infection. *Int. J. Otorhinolaryngol. Head. Neck. Surg* **2021,** *7*, 867; doi: 10.18203/issn.2454-5929.ijohns20211583.
32. Moorthy, A.; Gaikwad, R.; Krishna, S.; Hegde, R.; Tripathi, K.K.; Kale, P.G.; et al. SARS-CoV-2, Uncontrolled Diabetes and Corticosteroids—An Unholy Trinity in Invasive Fungal Infections of the Maxillofacial Region? A Retrospective, Multi-centric Analysis. *J. Maxillofac. Oral. Surg* **2021,** *20*, 1–8; doi: 10.1007/s12663-021-01532-1.
33. Hanley, B.; Naresh, K.N.; Roufosse, C.; Nicholson, A.G.; Weir, J.; Cooke, G.S.; et al. Histopathological findings and viral tropism in UK patients with severe fatal COVID-19: a post-mortem study. *Lancet. Microbe* **2020,** *1*, e245–53; doi: 10.1016/S2666-5247(20)30115-4.
34. Dallalzadeh, L.O.; Ozzello, D.J.; Liu, C.Y.; Kikkawa, D.O.; Korn, B.S. Secondary infection with rhino-orbital cerebral mucormycosis associated with COVID-19. *Orbit* **2021**, 1–4; doi: 10.1080/01676830.2021.1903044.
35. Placik, D.A.; Taylor, W.L.; Wnuk, N.M. Bronchopleural fistula development in the setting of novel therapies for acute respiratory distress syndrome in SARS-CoV-2 pneumonia. *Radiol. Case. Reports* **2020,** *15*, 2378–81; doi: 10.1016/j.radcr.2020.09.026.

36. Bellanger, A-P.; Navellou, J-C.; Lepiller, Q.; Brion, A.; Brunel, A-S.; Millon, L.; et al. Mixed mold infection with Aspergillus fumigatus and Rhizopus microsporus in a severe acute respiratory syndrome Coronavirus 2 (SARS-CoV-2) patient. *Infect. Dis. Now* **2021**, S2666–9919(21)00030-0; DOI: 10.1016/j.idnow.2021.01.010.
37. Sungurtekin, H.; Sargin. F.; Akbulut, M.; Karaduman, S. Severe Rhinocerebral Mucormycosis Case Developed after COVID-19. *J. Bacteriol. Parasitol* **2021**, *12*, 386; Available from: https://www.longdom.org/open-access/severe-rhinocerebral-mucormycosis-case-developed-after-covid19-61170.html
38. Pakdel, F.; Ahmadikia. K.; Salehi. M.; Tabari, A.; Jafari, R.; Mehrparvar, G.; et al. Mucormycosis in patients with COVID-19: A cross-sectional descriptive multicenter study from Iran. *Mycoses* **2021,** 10.1111/myc.13334; DOI: 10.1111/myc.13334.
39. Buil, J.B.; van Zanten, A.R.H.; Bentvelsen, R.G.; Rijpstra, T.A.; Goorhuis, B.; van der Voort, S.; et al. Case series of four secondary mucormycosis infections in COVID-19 patients, the Netherlands, December 2020 to May 2021. *Euro. Surveill* **2021**, *26*, 2100510; DOI: 10.2807/1560-7917.ES.2021.26.23.2100510.
40. Bayram, N.; Ozsaygılı, C.; Sav, H.; Tekin, Y.; Gundogan, M.; Pangal, E.; et al. Susceptibility of severe COVID-19 patients to rhino-orbital mucormycosis fungal infection in different clinical manifestations. *Jpn. J. Ophthalmol* **2021,** *65*, 515–525; doi: 10.1007/s10384-021-00845-5.
41. Rabagliati, R.; Rodríguez, N.; Núñez, C.; Huete, A.; Bravo, S.; Garcia, P. COVID-19–Associated Mold Infection in Critically Ill Patients, Chile. *Emerg. Infect. Dis* **2021**, *27*, 1454; DOI: 10.3201/EID2705.204412.
42. Bhavani, P.N.; Patil, V.; Patil, S.D. Imaging findings of Rhino-Orbito-Cerebral Mucormycosis in a COVID-19 patient. *Eurorad*, **2021**, 17205; Accessed on 24 August 2021 [Online]. Available from: https://www.eurorad.org/case/17205.
43. Khan, N.; Gutierrez, C.G.; Martinez, D.V.; Proud, K.C. A case report of COVID-19 associated pulmonary mucormycosis. *Arch. Clin. Cases* **2020,** *7*, :46–51; doi: 10.22551/2020.28.0703.10172.
